# A Case of Rosai-Dorfman Disease Presenting as an Isolated Pleural Plaque

**DOI:** 10.7759/cureus.41334

**Published:** 2023-07-03

**Authors:** Arun Rathinam, Sushan Gupta, Rukhsaar Khanam, Tanmay Sahai

**Affiliations:** 1 Internal Medicine, Carle Foundation Hospital, Urbana, USA; 2 Hematology and Oncology, Carle Foundation Hospital, Urbana, USA

**Keywords:** extranodal rdd, non-langerhans cell histiocytosis, pleural plaque, emperipolesis, pleural mass, rosai-dorfman disease

## Abstract

Rosai-Dorfman disease (RDD) is a rare non-Langerhans histiocytic disorder primarily involving lymph nodes. Extranodal RDD has a heterogenous presentation, and isolated pulmonary involvement is rare. We report the only case of RDD presenting as an isolated pleural mass. Our patient was a 55-year-old female with multiple comorbidities who presented with chest pain. Imaging revealed an enlarging pleural-based lesion. She underwent resection of the pleural mass, showing an atypical histiocytic infiltrate in a prominent background of collagenous fibrosis. Immunohistochemistry shows CD1a-negative and S100-positive atypical histiocytic cells demonstrating emperipolesis, confirming the diagnosis of RDD. She is currently on six-month CT surveillance with no recurrence of the disease. This case highlights the unique pulmonary presentation of RDD. It also underscores that observations may be appropriate in isolated asymptomatic pleural involvement cases.

## Introduction

Rosai-Dorfman disease (RDD), also known as Rosai-Dorfman-Destombes disease, is a rare non-Langerhans cell histiocytic disorder characterized by the accumulation of CD68-negative, S100-positive, and CD1a-negative histiocytes [1]. The classic nodal RDD commonly presents as bilateral lymphadenopathy with a preponderance for males and patients of African descent [2]. The extranodal presentation can be seen in approximately 43% of cases [1,2]. Cutaneous RDD is the most common extranodal form of RDD; however, in rare cases, the paranasal sinuses, salivary glands, and upper and lower respiratory tract can also be involved [1,2]. Isolated pleural involvement is extremely rare in RDD, making the diagnosis challenging in these cases. We present the case of a middle-aged patient who presented with isolated pleural plaque diagnosed later as RDD.

## Case presentation

**Figure 1:**
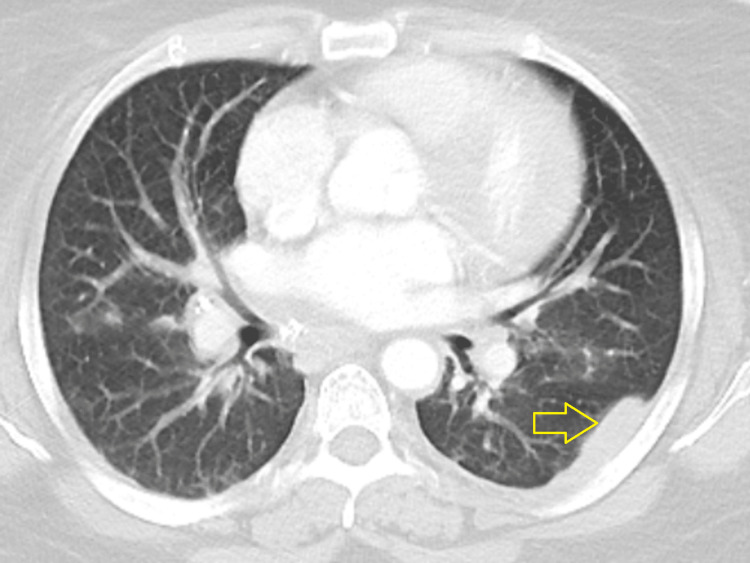
CT chest with IV contrast showing left pleural-based lesion (yellow arrow) along the superior segment of the left lower lobe.

Our patient was a 55-year-old Caucasian female with a medical history significant for type 2 diabetes mellitus on insulin (last A1c level obtained two weeks prior was 8.0%), hypertension, peripheral arterial disease, hyperlipidemia, and pulmonary embolism on rivaroxaban, and she was admitted to the hospital for constant left-sided chest pain for two days. Her condition worsened with exertion. The patient also complained of a cough for three days before admission. The review of symptoms was negative for constitutional symptoms, weight gain, leg swelling, shortness of breath, and orthopnea. She reported smoking half a pack of cigarettes a day until 2013, with a total of 20 pack-year smoking history. The patient denied any significant travel or family history. Her medications on admission included aspirin, atorvastatin, lisinopril, metoprolol tartrate, rivaroxaban, and insulin. On admission, the patient was febrile with temperature of 37.8 °C (100 °F), hemodynamically stable with blood pressure in 160s systolic, tachycardic with a heart rate of 130 beats per minute, and maintaining oxygen saturation >94% on room air. Significant labs on admission included elevated white blood cell count (16480/cu.mm) with neutrophilic dominance (13850/cu.mm) and elevated C-reactive protein (CRP) at 1.10 mg/dl (reference range -0.00 to 0.29 mg/dl). The rest of the labs, including serum chemistry, troponin, brain natriuretic peptide (BNP), procalcitonin, and D-dimer, were normal. A chest X-ray on admission showed pleural thickening/mass at the left chest wall. A review of imaging revealed nonspecific pleural thickening along the left chest wall on CT angiography done two years back. A repeat contrast-enhanced CT in the current admission was performed, which showed worsening of the pleural-based lesion, now extending along the pleural surface of the superior segment of the left lower lobe (Figure 1).

**Figure 2:**
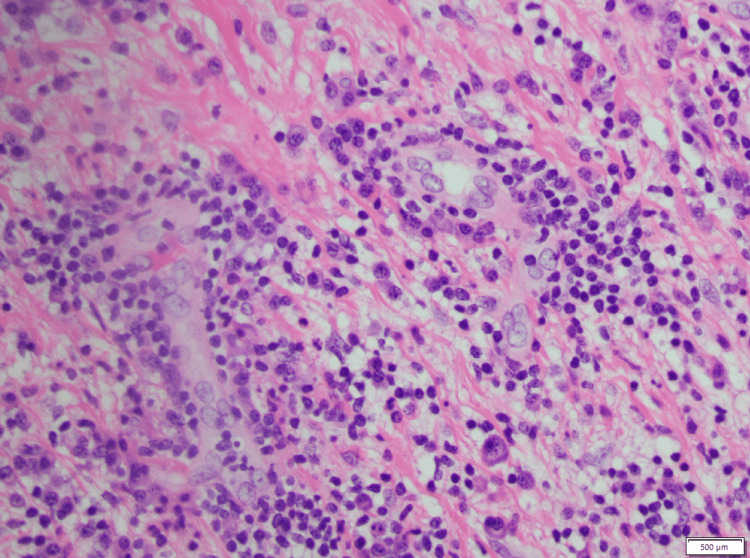
Histopathology showing atypical histiocytes embedded in a background of collagenous fibrosis with admixed plasma cells and lymphocytes

The CRP levels normalized one month later. The patient was scheduled for an outpatient fluoroscopy-guided biopsy of the lesion but was lost to follow-up. She was admitted again a couple of months as an inpatient and underwent successful resection of the pleural mass by cardiothoracic surgery. The patient had an uneventful recovery. Initial histopathology suggested sclerosed fibrosis mixed with inflammatory infiltrate consisting of atypical histiocytes, plasma cells, and lymphocytes (Figure 2). The atypical lymphocytes also demonstrated round nuclei with a single, centrally located prominent nucleolus and abundant clear cytoplasm.

**Figure 3:**
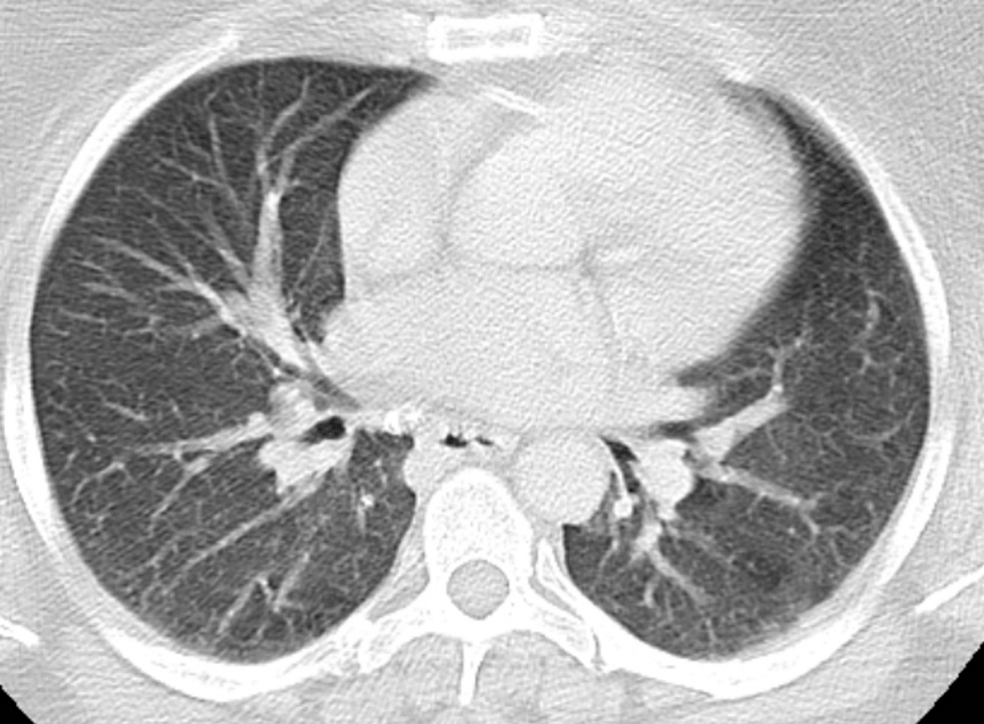
Repeat CT after resection of the mass showing no radiographic disease

The histopathology slides were sent to Mayo Clinic for a second opinion, where the diagnosis of RDD was confirmed. The atypical histiocytic cells that showed emperipolesis stained positive for S100 and negative for CD1a. The plasma cells did not exhibit an increased IgG4:IgG ratio. The patient also underwent a PET-CT and MRI brain, which ruled out systemic involvement from RDD. The patient was managed conservatively after the surgical removal of the mass. She underwent a repeat CT chest without contrast five months later, which showed a bandlike opacity in the left lower lobe, likely scarring, and she was continued on routine surveillance with another follow-up CT scan six months later that showed no disease recurrence. The area of prior pleural thickening was also unremarkable in the subsequent CT images (Figure 3).

The patient has had no recurrence of symptoms and is currently on routine surveillance with follow-up CT imaging every six months.

## Discussion

RDD is a rare histiocytic disease with an estimated prevalence of 1:200000 in the United States [3]. Extranodal presentation of RDD is heterogenous, commonly presenting with concomitant nodal involvement [3]. Isolated pulmonary involvement is seen in only approximately 2% of cases, with only one additional report describing isolated pleural involvement with pleural effusion in RDD [4]. To the best of our review, this is the only case report that described RDD presenting as an isolated asymptomatic pleural mass without any pleural effusion.

The pathogenesis and origin of RDD are still unclear. Initially, it was believed to be polyclonal in origin, presenting independently or in association with malignancies, such as Hodgkin and non-Hodgkin lymphoma; immune-mediated conditions, such as systemic lupus erythematosus; and juvenile arthritis [3]. More recent studies have demonstrated nodal and extranodal RDD clonality, with mutations in the NRAS, KRAS, MAP2K1, and ARAF genes. Moreover, germline mutations in SLC29A3 have been demonstrated with familial RDD [5,6]. Various viral infections have also been studied as predisposing agents for RDD; however, the causal association has not been demonstrated [7-9]. Pulmonary involvement in RDD has been described previously with presenting symptoms varying from interstitial lung disease, pulmonary nodules, and tracheobronchial involvement [3]. Our patient presented with a pleural mass that eventually progressed in size. This is a rare presentation of RDD. Another isolated case of pleural involvement in RDD describes an 81-year-old female who presented with shortness of breath and was found to have large loculated pleural effusion with pleural thickening [4]. The identification of RDD can be challenging in these cases, and differentials, such as malignancy, sarcoidosis, vasculitis, mycobacterial, and fungal infections, need to be ruled out. The diagnosis can usually only be confirmed with a biopsy of these lesions. Moreover, as in our case, extra nodal forms tend to present with extensive fibrosis and scant emperipolesis, further posing a diagnostic challenge [1]. In these cases, immunohistochemistry becomes vital, especially to rule out other histiocytic disorders, such as Langerhans cell histiocytosis and malignant histiocytosis. RDD is characteristically S100 and CD68 positive and CD1a negative, with an S100 stain highlighting the extent of emperipolesis [1]. Extranodal RDD can involve the skin (10%), nasal cavity (11%), bone (5%-10%), orbital tissue (11%), and central nervous system (5%). The prognosis in such cases depends on the number of extranodal sites involved [1]. Other case reports described patients with pleural effusions and had concomitant involvement of other serosal surfaces, such as the epicardium and mesentery [10,11]. Our patient presented with isolated pleural involvement, with MRI and PET scans ruling out other organ system involvement. There is no uniform approach regarding whether treatments should be initiated in cases where resection led to a radiographic negative disease state. One study showed 50% remission in patients with uncomplicated lymphadenopathy spontaneously or after surgical resection in nodal RDD [12]. In our patient, after the initial resection of the pleural mass for the diagnoses, we have not noticed a relapse on the six-month follow-up CT imaging for the last two years. However, systemic corticosteroid therapy should be considered when local recurrences are identified after resection [4,13].

## Conclusions

RDD can have a heterogeneous pulmonary presentation, including isolated pleural involvement, like in our case. The diagnosis of extranodal RDD is challenging, often needing biopsy with immunohistochemistry testing. Isolated asymptomatic pleural thickening in RDD may not warrant systemic treatment or surgery, and such patients can be managed conservatively with regular surveillance.
